# Acute unilateral vestibulopathy and corticosteroid treatment - A randomized placebo-controlled double-blind trial

**DOI:** 10.1177/09574271241307649

**Published:** 2024-12-18

**Authors:** Julia Sjögren, Per-Anders Fransson, Måns Magnusson, Mikael Karlberg, Fredrik Tjernström

**Affiliations:** 1 Department of Clinical Sciences, Otorhinolaryngology Head and Neck Surgery, Skåne University Hospital, 5193 Lund University, Lund, Sweden

**Keywords:** vestibular neuritis, acute unilateral vestibulopathy, corticosteroid treatment, steroid treatment, randomized placebo-controlled trail

## Abstract

**Background:**

The efficacy of corticosteroids for treating acute vestibular neuritis, or acute unilateral vestibulopathy (AUVP), remains controversial.

**Objective:**

This study aimed to evaluate whether corticosteroids improve vestibular function and reduce symptoms in both acute and chronic phases of AUVP.

**Methods:**

This randomized, placebo-controlled, double-blind trial included patients with AUVP (ages 18–80) from emergency departments at three sites in southern Sweden. Patients were randomly assigned to one of three groups: placebo, 3-day or 10-day corticosteroid treatment. The steroid groups received intravenous betamethasone followed by oral steroids, while the placebo group received intravenous saline followed by oral placebo. The primary outcome was canal paresis (%) after 12 months, measured via caloric testing. Secondary outcomes included vHIT gain, Diary Vertigo score, Dizziness Handicap Inventory, and Hospital Anxiety and Depression Scale. Analyses were conducted on an intention-to-treat basis. The trial was registered with the EU Clinical Trials Register (EudraCT Number: 2014-005484-32) and ClinicalTrials.gov (NCT00802529).

**Results:**

From December 2015 to March 2021, 350 patients were screened, and 69 were included: 23 in the 10-day corticosteroid group, 22 in the 3-day corticosteroid group, and 24 in the placebo group. All groups showed significant improvement in caloric function over time (*p* = .002), with no significant differences between groups at any time point (*p* = .629). Change in caloric asymmetry from baseline to 12 months did not differ between the treatment groups and the placebo group: mean difference −8.34 (95% CI –25.93 to 9.26; *p* = .347) in the 10-day steroid group and −6.61 (−24.67 to 11.45; *p* = .467) in the 3-day steroid group, compared with placebo. Secondary outcomes showed no significant differences between groups. Corticosteroid treatments were well tolerated with no safety concerns.

**Conclusions:**

Corticosteroid treatment does not significantly improve caloric recovery, vHIT gain recovery, or subjective well-being in patients with AUVP.

## Introduction

Acute unilateral vestibulopathy (AUVP) commonly known as vestibular neuritis,^1^ is due to a sudden loss of peripheral vestibular function, resulting in static and dynamic effects on postural stability and oculomotor function. The acute effects include spontaneous nystagmus, head and ocular tilt, postural disequilibrium, altered vestibulo-ocular and spinal reflexes.^2^ In most patients, the perceived symptoms significantly subside within a few days and continue to gradually resolve over subsequent weeks. However, the loss of dynamic reflexes often persists and remains functionally inadequate and asymmetric, as complete restitution of vestibular function rarely occurs.^3^ Improvement from the condition, involves a combination of restitution of peripheral vestibular function and gradual central vestibular compensation.^4^

Whether corticosteroids enhance vestibular functional recovery or not has been investigated with conflicting results.^5–9^ In the largest double-blind RCT study, it was shown that corticosteroids improved caloric responsiveness.^8^ Other studies have either not shown any beneficial aspect of corticosteroid treatment^9^ or suggested that treatment might accelerate recovery although the long-term performance was not impacted.^6^ Observations have also highlighted critical time frames for treatment initiation for AUVP^10^ similar when treating Bell’s palsy with corticosteroids,^11^ a condition that may have the same pathogenesis. A Cochrane review in 2011 concluded that there was insufficient evidence to recommend corticosteroid treatment^12^ and advised that future studies combined subjective assessments with evaluation of vestibular function. However, a more recent meta-analysis in 2018^13^ found that steroid treatment in vestibular neuritis offered a statistically significant benefit compared to control therapies.

The primary objective of this study was to evaluate the short and long-term effects of steroid treatment for AUVP, both assessing vestibular function and subjective symptoms. The aim with this integrated approach was to provide a comprehensive understanding of the impact of steroid treatment on patients' vestibular function and overall well-being.

## Methods

We conducted a randomized, double-blind, placebo-controlled trial from December 1, 2015, through March 1, 2021. Patients (18–80 years of age) were recruited from the emergency department at three sites (dept. of OtoRhinoLaryngology Head and Neck Surgery, Skåne University Hospital, dept. of OtoRhinoLaryngology Helsingborg, dept of OtoRhinoLaryngology Kristianstad) in southern Sweden. The trial protocol, available at clinicaltrials.gov and EUdraCT, was approved by the regional scientific ethics committee. The trial was performed in accordance with the principles of the Declaration of Helsinki (Dnr 2015/5, EPN, Lund University, Sweden). Written informed consent was obtained from all participants prior to enrollment in the study.

The diagnosis of AUVP was based on the following criteria: a history of acute onset of vertigo without auditory or neurological symptoms and definite unilateral vestibulopathy. This included spontaneous, contralesional, horizontal-torsional nystagmus that did not change direction with gaze and increased without visual fixation, as well as an ipsilesional pathologic head impulse test. Additionally, there were no acute central neurological signs, such as central ocular motor or vestibular signs, pronounced skew deviation, gaze-evoked nystagmus, or acute audiologic or otologic signs.^14^ This meets the criteria set by the Bárány Society,^1^ except for the duration of more than 24 h, of the symptoms but adhere with term “AUVP in evolution” of the same classification. However, patients that during the study period developed an episodic disease were excluded from the study. The subjects had to be capable of making their own decisions and were excluded if they had a history of vertiginous disease or had symptoms that began more than 48 h before possible inclusion in the study. Complete lists of inclusion and exclusion criteria are provided in Table 1.

**Table 1.** Inclusion/exclusion criteria.

Inclusion criteria	Age between 18 and 80
	Acute vestibular syndrome^a^
	No dangerous HINTS^a^
	Disease onset <48 hours
Exclusion criteria	New symptoms from tinnitus or hearing loss
	Pregnancy
	Previous gastric ulcer
	Significant psychiatric disorder^b^
	Blood pressure >180 systolic and/or >110 diastolic
	Glaucoma
	Serious infection or neutropenia
	Chronic otitis media^c^
	Not fluent in Swedish^d^
	Incapable of making an informed decision

^a^**H**ead **I**mpulse test, **N**ystagmus, **T**est of **S**kew - see reference 15.

^b^That is, not excluding SSRI treatment of mild depression.

^c^Making caloric irrigation impossible (air irrigation not the same stimulus).

^d^Not possible to fill out questionnaires nor reading the written information.

### Randomization and treatment

Participants were randomly assigned to one of three treatment groups in blocks of six: a placebo group, a 3-day steroid treatment group or a 10-day steroid treatment group. The intravenous treatment was blinded to both patient and doctor throughout the study period and the code was only broken after all assessments were done. Only the emergency nurse responsible for the injection had any information but was not involved further in the study. On day one, the 10-day steroid treatment group and the 3-day steroid treatment group received an injection of intravenous betamethasone 8 mg (equivalent to 50 mg prednisone and 8 mg dexamethasone). The placebo group received a matching infusion of sodium-chloride in the emergency room. In the 10-day steroid treatment group 50 mg of prednisolone (equivalent to 50 mg prednisone) was administered daily as a single morning dose on days two through six, tapering the dose of 10 mg/day on days seven through ten and completing the treatment on day 11 with a dose of 5 mg. The 3-day steroid treatment group received a dose of 50 mg prednisolone on day two through three, followed by a matching dose of placebo on days four through 11. The placebo group received matching doses of placebo medication on days two through 11.

On inclusion all the patients were provided with the study medicine for the following ten days in prepacked, standardized cannisters with written instructions. Most participants were admitted to the hospital for at least 1 day and discharged when they felt they could manage on their own at home. Compliance was assessed at follow-up after 1 month.

Participants received 20 mg of Omeprazole (proton-pump inhibitor) once a day on day one through 14 to reduce the risk of gastric ulcers increased by a possible steroid treatment.

Antiemetic medication was administered if needed during the initial 2 weeks, with instructions for subjects to take the lowest possible dose to facilitate the process of vestibular compensation. All participants were informed about the importance of vestibular exercise, received verbal and written instructions on how to preform vestibular exercise and instructed to initiate the exercise as soon as possible (directly or the following day).

All patients kept a structured diary for the first 4 weeks, assessing their symptoms, need for antiemetic medication and adverse events. Patients were assessed within a week, one, three, and 12 months after inclusion.

## Outcomes

### Primary outcome

The primary outcome was unilateral vestibular loss measured as vestibular asymmetry in percent upon bithermal caloric testing. The patient was reclined with their head at a 30° angle and fitted with infrared video oculography goggles (Interacoustics, Visual Eyes) that closed out all light to avoid fixation. The mean peak slow-phase velocity of the nystagmus during caloric irrigation of both ears (R denotes right, and L left) with water at 30°C and 44°C was measured. An asymmetry between the two ears of more than 22 % as measured with the use of Jongkees’s formula ([(R30° + R44°) - (L30° + L44°)] ÷ (R30° + R44° + L30° + L44°)) * 100 for vestibular paresis was considered abnormal.^15^

### Secondary outcomes

Vestibular function was also assessed with the video head impulse test (vHIT), that is, the ability of the vestibulo-occular reflex (VOR) to maintain the eye position on a visual reference point during fast accelerations and decelerations of the head, using the Interacoustics (EyeSeeCam version 1.2, Interacoustics A/S, Middelfart, Denmark),^16^ ICS Impulse video goggles, Otometrics, Taastrup, Denmark^17^ and SYNAPSIS.^18^ Vestibular gain measurements were obtained for all six semicircular canals. However, given that the video head impulse test demonstrates the highest precision for horizontal values, we exclusively present data from the lateral semicircular canals.^19^ The vHIT test procedure is well defined.^17^ In short, patients were seated 1.5 m in front of a wall with a small target fixed at eye level. The subject was instructed to always keep visual fixation on the target during the assessment. All head impulse tests were carried out by a few experienced examiners who stood behind the subject, imposing rapid, unpredictable head rotations in the plane of all six semicircular canals with peak velocities exceeding 150^◦^/s, accelerations/decelerations chiefly within 3000–8000°/s^2^ and a movement amplitude of about 10–25°. The vHIT testing continued until the software had accepted at least 10 head impulses in each direction according to the criteria above. Finally, the software algorithm was employed to calculate the gain (eye movement/head movement) average for each direction. For patients assessed using the Interacoustics software, the regression gain was utilized in the conclusive analysis, while for those examined with Otometrics and SYNAPSIS software, the gain was computed based on the area under the curve (50–150 ms) and was subsequently employed. The gain value was a secondary outcome, and an abnormal gain considered to be <0.8.^20^

Upon inclusion all patients were instructed to keep a structured diary and score their symptoms of vertigo from day one to day 28 on a Likert scale (1 = no symptoms and 10 = worst possible symptoms).

Subjective symptoms were followed up with two different questionnaires after three and 12 months after inclusion: Dizziness Handicap Inventory (DHI) and Hospital Anxiety and Depression scale (HADS).

The Dizziness Handicap Inventory determines how often participants have dizziness-related difficulty or distress in 25 situations, each on a scale that ranges from 0 (no distress) to 4. The overall score is the sum of all the responses; scores range from 0 (no self-perceived dizziness handicap) to 100.^21^

The Hospital Anxiety and Depression Scale (HADS) is a 14-question enquiry frequently used to assess psychological distress in non-psychiatric patients. It consists of two subscales, Anxiety and Depression, with seven questions for each. Each question is scored between zero (no impairment) and three (severe impairment), with a maximum score of 21 for anxiety or depression.^22^ Swedish versions of HADS and DHI are validated for use in clinical settings.

**Figure 1.**
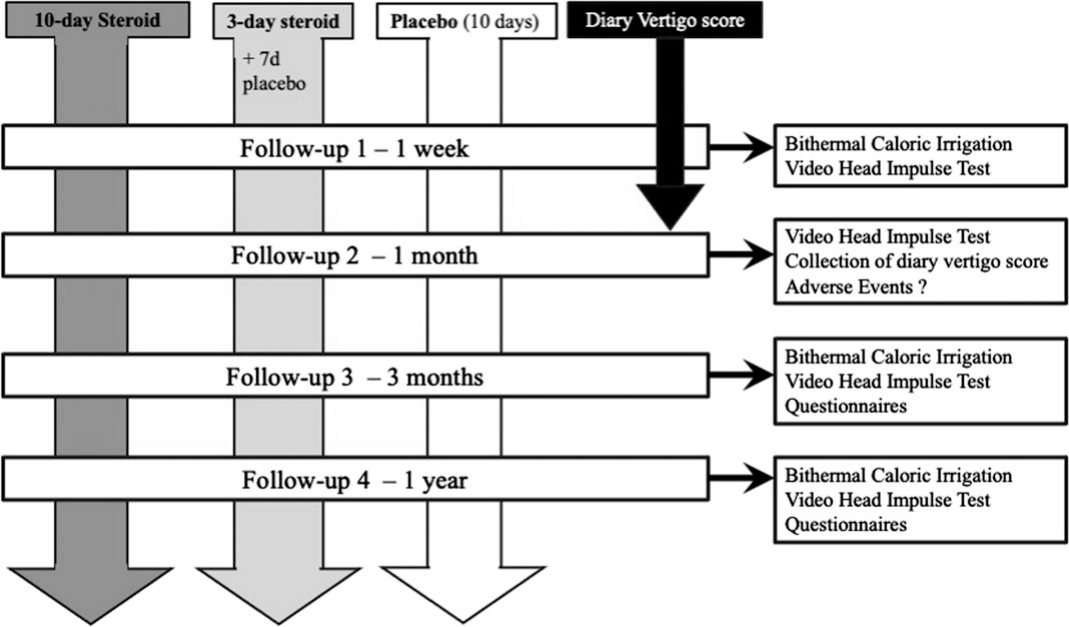
Study design and follow-up.

Baseline evaluation with bithermal caloric testing and vHIT were carried out as soon as possible after inclusion (mean 4 days, range 1–9 days). Follow-ups were performed after 1 month (vHIT), 3 months (calorics, vHIT, and questionnaires), and 1 year (calorics, vHIT, and questionnaires) (Figure 1).

### Adverse events

All patients received written information about possible adverse effects of steroids. They were instructed to write down any adverse and/or side effects in the diary. Adverse effects of the medication were assessed at follow-up after 1 month, 3 months, and 1 year.

Treatment was stopped if patients did not want to continue the study.

## Statistical analysis

Power calculations were made for the primary end point (caloric responsiveness) and based on Strupp et al.^8^ Sample sizes were calculated using the GPower 3.1.9.7 software. The mean (±SD) difference requirements were set to 25 ± 26% (calculated with Jongkees’s formula).^8^ The power analysis yielded an effect size of 0.96 and a sample size requirement of 18 patients in each group, assuming a t-test for difference between two independent means (two groups), a two-sided alpha level of 0.05 and a statistical power of 0.80.

The baseline characteristics were compared between the treatment groups with the use of chi-square test for dichotomous parameters and the Mann-Whitney U-test for continuous parameters.

All analyses were performed in the modified intention-to-treat population. The parameters canal paresis values, the ipsilesional vHIT gains, the diary vertigo scores, and the sores from DHI and HADS were analyzed using repeated measures GLM ANOVA (General Linear Model Analysis of Variance).^23^ The main factors and factor interactions analyzed were Treatment [10-day steroid treatment versus 3-day steroid treatment versus placebo; d.f. 2] and Time [(1 week vs 3 months vs 12 months; d.f. 2 for canal paresis value); (1 week vs 1 month vs 3 months vs 12 months; d.f. 3 for vHIT gain); (3 months vs 12 months; d.f.1 for the DHI, HADS-anxiety score, and HADS-depression score); and (day 1 to day 28; d.f. 27) for the diary vertigo score]. For the GLM ANOVA, two-sided *p-*values <.05 were considered significant.

For the DHI and HADS outcomes, log-transformed data was utilized in the repeated measures GLM ANOVA to achieve a more symmetric distribution, enhancing the validity of the analysis.

Non-parametric post-hoc tests were used in all statistical evaluations since the Shapiro-Wilk test revealed that some parameters were not normally distributed, and normal distribution could not be obtained by log-transformation. The Mann-Whitney U-test was used for between group post-hoc comparisons, that is, analyzing the effect of the treatment. The Wilcoxon matched-pairs signed-rank test (exact sig. 2-tailed) was used for within-group, that is, analyzing the recovery in each group over time. In the analyses, *p*-values < .025 were considered significant for canal paresis values, *p*-values < .0125 for ipsilesional vHIT gain, and *p*-values < .05 for the HADS and DHI questionnaires, after the *p*-values had been adjusted using the Bonferroni correction. Analyses were performed with the SPSS software, version 28.0 (IBM SPSS Statistics).

As a secondary consideration with regards to clinical practice primary and secondary functional outcome was dichotomized into recovery or not and analyzed using the chi-square test. 

## Results

**Figure 2.**
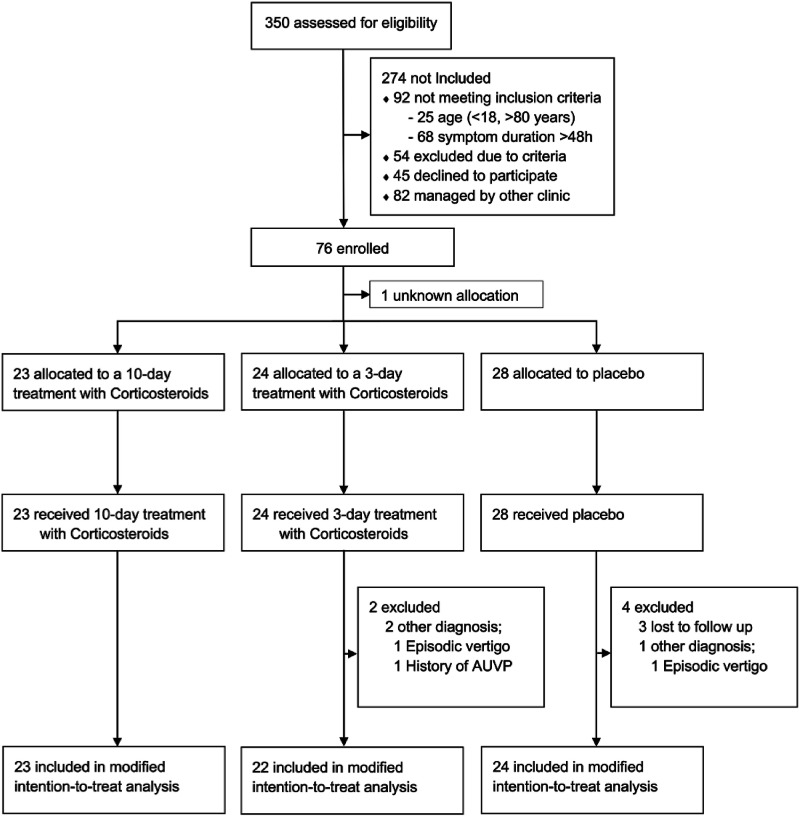
Consort flow-chart of the modified intention-to-treat population.

Between December 1, 2015, and March 1, 2021, 350 patients were screened of which 76 met the criteria in time frame and diagnosis for inclusion and were inclined to participate. The allocation key for one patient was lost, rendering their group assignment unknown; as a result, the patient was excluded from the analysis. 28 were randomly assigned to the placebo group, 23 to the 10-day steroid treatment group and 24 to the 3-day steroid treatment group. Four subjects in the placebo group and two in the 3-day steroid treatment group were excluded due to evolving an episodic vertigo disease or lost to follow-up (no follow-up data) (Figure 2). No patients discontinued the treatment. The final groups in the modified intention-to-treat population became thus, 24 subjects in the placebo group, 23 in the 10-day steroid treatment group and 22 in the 3-day steroid treatment group, making a total of 69 subjects used in the final analyses. The three groups were balanced with the respect to baseline characteristics (Table 2).

**Table 2.** Baseline characteristics of the modified intention-to-treat population.

	Steroids 10 days (*N* = 23)	Steroids 3 days (*N* = 22)	Placebo (*N* = 24)
Age (years)	56.8 ± 13.9	53.9 ± 13.3	55.7 ± 13.7
min–max	24–75	22–72	26–78
Sex			
Female	8 (35%)	6 (27%)	10 (42%)
Male	15 (65%)	16 (73%)	14 (58%)
Lesioned ear			
right	12 (52%)	12 (55%)	12 (50%)
left	11 (48%)	10 (45%)	12 (50%)
Onset to treatment (hours)	11.4 ± 9.7	19.5 ± 10.4	17.2 ± 12.9
min–max	4–47	7–46	3–47
Onset to examination (days)	3.2 ± 1.9	3.6 ± 2.0	4.1 ± 1.7
min–max	1–9	1–8	2–9
Caloric Asymmetry (%)	63 ± 35	60 ± 33	64 ± 38
min–max	0–100	0–100	3–100
Ipsilesional gain	0.52 ± 0.32	0.56 ± 0.28	0.53 ± 0.24
min–max	0.14–1.02	0.24–1.04	0.14–0.94
Diary vertigo score (1–10)	8.7 ± 1.7	8.5 ± 1.7	8.3 ± 2.3
min–max	4–10	4–10	3–10

Plus–minus values are means ±SD.

**Figure 3.**
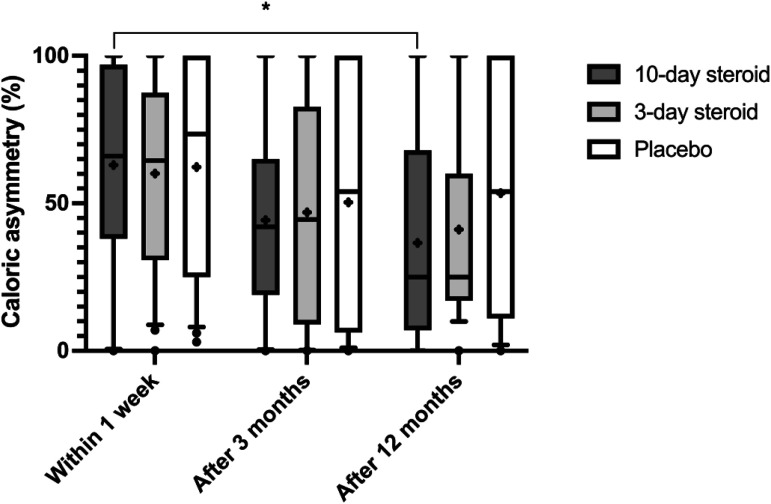
Caloric asymmetry in the three groups. In the box representing each treatment group, the symbol “x” denotes the mean, the horizontal lines represent the 25th, 50th (median), and 75th percentiles, while the error bars above and below the boxes indicate the 10th and the 90th percentiles. Significant *p*-values after Bonferroni correction are presented *= <0.025.

The repeated measures GLM ANOVA revealed a significant overall reduction in caloric asymmetry over time (*p* = .002; Table 3). Change in caloric asymmetry from baseline to 12 months did not differ between the treatment groups and the placebo group: mean difference −8.34 (95% CI –25.93 to 9.26; *p* = 0.347) in the 10-day steroid group and −6.61 (−24.67 to 11.45; *p* = 0.467) in the 3-day steroid group, compared with placebo (Figure 3). Notably, in the 10-day steroid group, caloric asymmetry significantly decreased from 63 ± 35% to 37 ± 35% between the initial examination (1 week) and 12 months (*p* = .014; Figure 3), while the 3-day steroid group showed a trend toward reduction, from 60 ± 33% to 41 ± 34% (*p* = .039; Figure 3). In contrast, the placebo group showed no significant change, with asymmetry shifting from 64 ± 38% to 54 ± 38% (*p* = .496; Figure 3).

**Table 3.** Repeated measures GLM ANOVA.

	Time	Treatment	Time^a^ Treatment
Caloric asymmetry	**0**.**002 [11**.**13**]	0.629 [0.47]	0.322 [1.16]
Ipsilesional vHIT gain	**<0**.**001 [57**.**94]**	0.687 [0.38]	0.732 [0.31]
Diary vertigo score	**<0**.**001 [277**.**24]**	0.524 [0.66]	**0**.**013 [4**.**76]**
DHI score	**0**.**006 [8**.**31]**	0.454 [0.81]	**<0**.**001 [9**.**49]**
HADS-anxiety score	0.126 [2.44]	0.526 [0.65]	0.251 [1.43]
HADS-depression score	0.078 [3.32]	0.286 [1.13]	0.431 [0.86]

Repeated measures GLM ANOVA with main factors “time” and “treatment (“10-day steroid treatment” vs “3-day steroid treatment” vs “placebo treatment”) and their factor interaction.

^a^F-values are presented within the squared parenthesis. Bold statistics denote *p*-values < .05.

**Figure 4.**
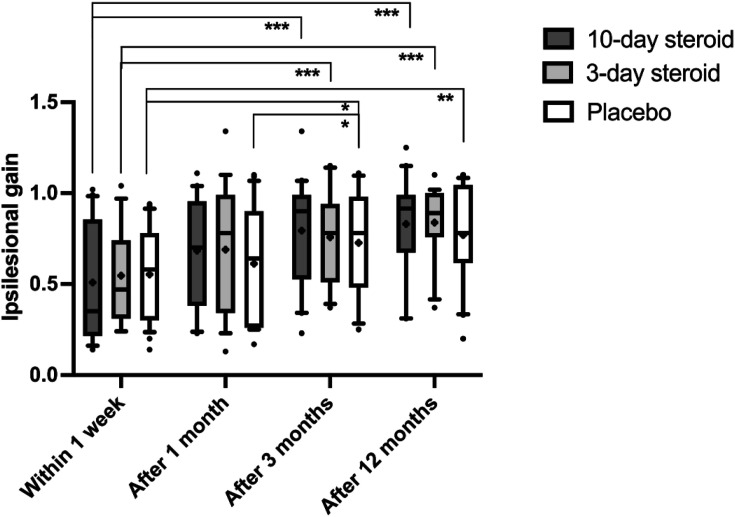
**Ipsilesional vHIT gain (lateral canal) in the three groups.** In the box representing each treatment group, the symbol “x” denotes the mean, the horizontal lines represent the 25th, 50th (median), and 75th percentiles, while the error bars above and below the boxes indicate the 10th and the 90th percentiles. Significant *p*-values after Bonferroni correction are presented *<.0125 ** <.01, *** <.001.

All groups exhibited progressive improvement in ipsilesional vHIT gain over time (*p* < .001; Table 3, Figure 4). The GLM ANOVA did not detect any significant treatment effect or interaction between treatment and time. Mann-Whitney post-hoc analysis also found no significant differences in vHIT gain recovery between groups (Figure 4). However, post-hoc Wilcoxon analysis revealed significant improvement in all groups between week one and 12 months, with the most pronounced enhancement in the 10-day corticosteroid group (*p* < .001; Figure 4) and in the 3-day corticosteroid group (*p* < .001; Figure 4).

Dichotomizing the functional outcome at the 12-month follow-up (<22% caloric asymmetry and/or vHIT gain >0.8) did not alter the finding that steroid treatment had no significant impact on vestibular function recovery in either primary or secondary outcomes (Table 4).

**Table 4.** Functional outcome after 12 months dichotomized.

	10-day Steroid (*n* = 23)	3-day Steroid (*n* = 22)	Placebo (*n* = 24)	*p*-values
Only a caloric recovery	0 (0%)	0 (0%)	1 (4%)	ns
Only a vHIT recovery	5 (22%)	7 (32%)	5 (21%)	ns
Both caloric and vHIT recovery	11 (48%)	10 (45%)	8 (33%)	ns
Neither caloric nor vHIT recovery	7 (30%)	5 (23%)	10 (43%)	ns

A caloric recovery is defined as <22% asymmetry and a vHIT recovery is defined as a gain >0.8.

**Figure 5.**
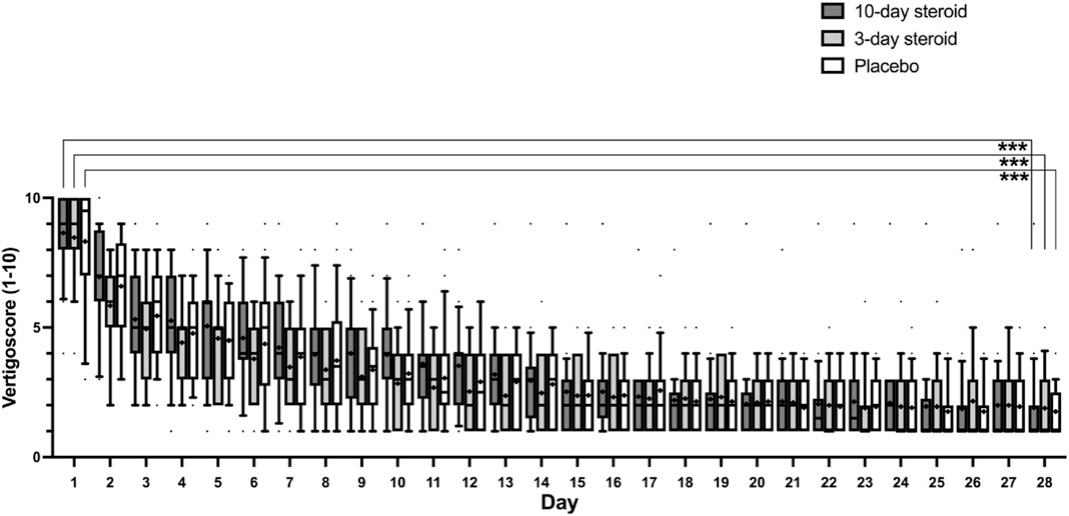
Diary of vertigo the first 4 weeks. In the box representing each treatment group, the symbol “x” denotes the mean, the horizontal lines represent the 25th, 50th (median), and 75th percentiles, while the error bars above and below the boxes indicate the 10th and the 90th percentiles. Significant *p*-values after Bonferroni correction are presented *<.05 ** <.01, *** <.001.

Vertigo symptoms, as measured by diary scores, decreased significantly in all groups during the first month: from 8.7 ± 1.7 to 1.9 ± 0.4 in the 10-day steroid group, 8.5 ± 1.7 to 1.9 ± 0.3 in the 3-day steroid group, and 8.32 ± 2.3 to 1.76 ± 0.9 in the placebo group. The repeated measures GLM ANOVA confirmed significant symptom relief across all groups (*p* < .001; Table 3) but found no significant treatment effect. A significant interaction between treatment and time was noted suggesting a slower vertigo score reduction over time in the 10-day steroid group compared to the 3-day and the placebo group (*p* = .013; Table 3). Post-hoc Mann-Whitney U-tests confirmed no significant differences between groups, while post-hoc Wilcoxon analysis showed significant symptom reduction from day one to day 28 in all groups (*p* < .001; Figure 5).

**Figure 6.**
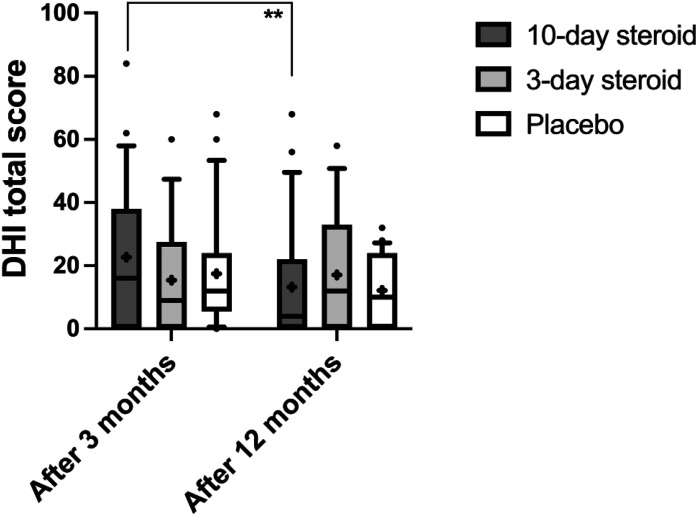
**Dizziness Handicap Inventory Score.** In the box representing each treatment group, the symbol “x” denotes the mean, the horizontal lines represent the 25th, 50th (median), and 75th percentiles, while the error bars above and below the boxes indicate the 10th and the 90th percentiles. Significant *p*-values after Bonferroni correction are presented **<.01.

For DHI and HADS, the repeated measures GLM ANOVA found no significant differences between groups (Table 3). In DHI, self-perceived dizziness handicap was significantly reduced at the 3- and 12-month follow-ups (*p* < .006; Table 3, Figure 6), with a significant interaction between treatment and time (*p* < .001; Table 3). Post-hoc Wilcoxon analysis showed significant score reductions after 12 months compared to 3 months in the 10-day group (Figure 6), though Mann-Whitney analysis revealed no differences between groups at either time point (Figure 6).

**Figure 7.**
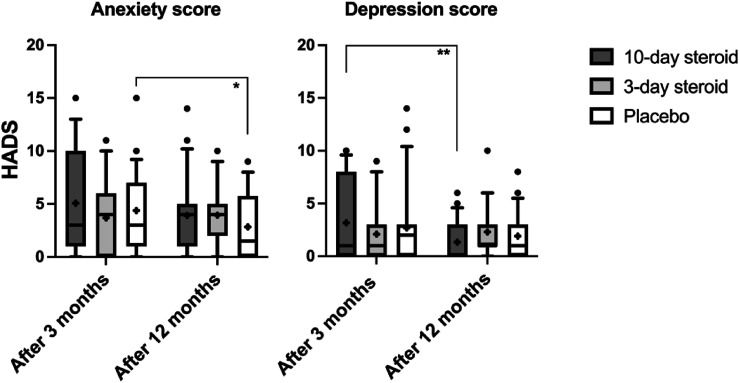
Hospital Anxiety and Depression Score. In the box representing each treatment group, the symbol “x” denotes the mean, the horizontal lines represent the 25th, 50th (median), and 75th percentiles, while the error bars above and below the boxes indicate the 10th and the 90th percentiles. Significant *p*-values after Bonferroni correction are presented *<.05.

HADS scores did not significantly decrease between the three and 12-month follow-ups for either anxiety or depression. There were no significant differences between groups or any significant interaction between treatment and time (Table 3). However, post-hoc analyses (Figure 7) indicated that placebo patients showed the greatest reduction in anxiety, while the 10-day treatment group significantly decreased their depression scores.

### Adverse events

No serious adverse events were reported in subjects treated with steroids. However, one patient experienced increased gastroesophageal reflux, while another reported stomachache, which could potentially indicate gastritis. None of the subjects displayed any signs of a gastric ulcer, but all were treated with omeprazole 20 mg daily the first 2 weeks

## Discussion

The findings suggest that administration of corticosteroids in patients with AUVP does not result in significant improvement of vestibular function or perceived symptoms neither in short nor long-term, which is in line with recent randomized controlled trials.^6,9^ According to our previous report,^10^ the timing from disease onset to steroid treatment seemed to impact the effectiveness in terms of caloric recovery, and affect length of hospital stay which is why the duration of symptoms (within 48 h) became one of our major inclusion criteria. Most patients included, received treatment within 24 hours. From all eligible patients 68, or close to 20%, could not be included because either the patients were admitted under a different diagnosis or just sought medical advice too late. We do believe that in conducting a trial the timing of treatment is important, and we theorized that the timing of treatment might have been the reason as to why some studies did not report a beneficial impact from corticosteroid treatment. That proved in our material not to be the case.

Although there was no significant difference in caloric recovery between the groups, post-hoc tests suggest a reduction in canal paresis after 1 year in patients treated with corticosteroids for 10 days, with a trend toward a similar reduction in those treated for 3 days, but not in the placebo group. This observation may indicate that corticosteroids influence caloric responsiveness, aligning with findings from the largest randomized controlled trial to date.^8^ However, a single significant *p*-value from a post-hoc test cannot match the statistical robustness of a GLM ANOVA analysis and should therefore be interpreted with caution. While the caloric test is valuable for diagnosing vestibular function, it has limitations when assessing the intricate process of vestibular compensation after AUVP.^24^ It primarily assesses the vestibular response to a low frequency stimulus, which does not necessarily correspond to the high-frequency head movements involved in daily life, that vHIT measures. The improvement of ipsilesional vestibular gain seemed to be unaffected by corticosteroid treatment, which corroborates the findings from the sole prior study that had explored this matter.^9^ However, vHIT does not necessarily correspond to subjective symptoms of dizziness.^25^To further investigate this, we included assessments of symptomatic improvement through diaries and questionnaires. Corticosteroid treatment did not reduce vertigo symptoms in the acute phase of AUVP nor did it reduce the self-perceived handicap caused by the vestibular loss in the chronic phase of the disease. Our results are in line with recent studies.^6,9^

One could argue that our inclusion criteria were too generous, since some patients displayed vHIT gain values within normal reference on the initial examination. On the other hand, the inclusion criteria were the same as set by the Bàràny Society and reflect the patients that we diagnose and treat in a clinical setting. Additionally, all patients initiated their treatment within 48 hours, a level of consistency that has been lacking in earlier studies and considered a potential confounder.

We did not include a no-treatment group which could be perceived as a confounder for the subjective outcome at least in the short-term. All patients received the same instructions with the same vestibular exercises with the aim to make the patient-doctor interaction identical in all cases.^26^ For the questionnaires, distributed after 3 and 12 months, the placebo-effect should be negligible.^26^ However, it is possible that the closely monitoring of the patients for the first 4 weeks constituted an increased placebo-effect and it is likewise certainly a fact that is it not the same to administer a drug as not to. It is thus wise to interpret our findings with care and counsel future patients that steroids may have a very small effect and avoid treatment whenever it could be harmful or doubtful. The primary focus in treating AUVP should be on instigating physiotherapy (vestibular rehabilitation) as soon as possible, where we do have moderate to strong evidence for its effectiveness.^27^ To get that message across to primary caregivers that are not neuro-otologists is of the utmost importance.

In conclusion, the findings suggest that steroid treatment does not improve symptoms in the acute or in the chronic phase of AUVP, nor does it enhance vestibular function in a major way. The treatment itself is harmless with the dosage administered in this study, despite the quite generous inclusion criteria.

## Statements and declarations
